# Adherence to antiretroviral therapy and factors affecting low medication adherence among incident HIV-infected individuals during 2009–2016: A nationwide study

**DOI:** 10.1038/s41598-018-21081-x

**Published:** 2018-02-16

**Authors:** Jungmee Kim, Eunyoung Lee, Byung-Joo Park, Ji Hwan Bang, Jin Yong Lee

**Affiliations:** 1 Medical Research Centre, Seoul National University College of Medicine, Seoul, Republic of Korea; 2 Department of Internal Medicine, Seoul National University College of Medicine, Seoul, Republic of Korea; 3 Graduate School of Public Health, Seoul National University, Seoul, Republic of Korea; 4 Department of Preventive Medicine, Seoul National University College of Medicine, Seoul, Republic of Korea; 5 Division of Infectious Diseases, Seoul National University Boramae Medical Centre, Seoul, Republic of Korea; 6 Public Health Medical Service, Boramae Medical Centre, Seoul National University College of Medicine, Seoul, Republic of Korea; 7 Institute of Health Policy and Management, Medical Research Centre, Seoul National University, Seoul, Republic of Korea

## Abstract

For ideal clinical benefit, human immunodeficiency virus (HIV)-infected individuals should receive continuous medication. This is the first nationwide antiretroviral therapy (ART) adherence study in Asia, where medication monitoring at national level is systemically available. We estimated the ART adherence of incident HIV-infected individuals and investigated factors affecting low medication adherence using the national health insurance (NHI) claims data from 2007 to 2016. Medication possession ratio (MPR) was used to measure medication adherence and risk factors were identified by multivariable logistic regression analysis. Of the 8,501 newly diagnosed HIV-infected individuals during 2009–2016 with at least one ART prescription, 70.4% of HIV patients had adequate adherence to ART defined as MPR ≥95%. Requiring prophylactic antibiotics, female gender, age of 0–19 and same or over 50 s compared to 30–39, and having a history of malignancy, lower socioeconomic status, not visiting tertiary hospital, and being diagnosed in the earlier years were risk factors for lower adherence (Odds ratio 1.7, 1.6, 1.6, 1.4, 1.6, 2.1, 1.2, and 1.6 to 3.8 respectively). Health authority should take into consideration of these modifiable and unmodifiable barriers to establish sustainable monitoring system at national level and to improve adherence.

## Introduction

The global target of the Joint United Nations Programme on human immunodeficiency virus (HIV)/acquired immunodeficiency syndrome (AIDS) (UNAIDS) to combat HIV infection by 2020 is summarized as the 90–90–90 target^1,2^. Since the innovative study by Granich in 2009, it has been recommended to initiate antiretroviral therapy (ART) as soon as an individual is diagnosed with an HIV infection, and early treatment is considered as the top priority in HIV management by clinicians^3^. A recent mathematical modelling study reported that interventions for HIV infection will impact the basic reproduction number and HIV incidence if they target HIV patients who are not yet undergoing ART rather than those who are already undergoing ART^4^.

An adherence to ART of 95% is required as an appropriate level to achieve maximal viral suppression^5–7^ and lower the rate of opportunistic infections^8^. Non-adherence is related to the development of ART resistance^9^, progression to AIDS^10^, and death^11^. However, in clinical practice, the maintenance of optimal ART adherence is challenging. A meta-analysis of 84 studies estimated that only 62% of HIV patients achieved optimal adherence (of >90%)^12^. Despite complete government funding of the medical service fees relevant to HIV (including ART) in Korea, there are limited data on medication adherence at the national level. In a setting in which patients have very low barriers to treatment, there may be risk factors for low ART adherence that have not been previously reported. In a former Korean study, continuity of care was assessed by analysing the consistency of hospital visits in 3- month intervals using a hospital-based HIV cohort of 247 patients who started ART between 2002 and 2008^13^. However, whether an individual is being treated with ART, and the continuity of medications are more valuable and direct indices than the number of clinic visits. Therefore, this study aimed to estimate the ART adherence of incident HIV-infected individuals and to investigate factors affecting low medication adherence in Korea.

## Results

### General characteristics of the study population

The annual number of HIV-infected individuals recruited from the national health insurance (NHI) cohort data using ART prescriptions was comparable to the actual number reported in the annual HIV incidence report of the Korea Centre for Infectious Disease Control and Prevention (KCDC). In Korea, public and private clinics mandatorily report all HIV-infected individuals to the KCDC.

**Figure 1:**
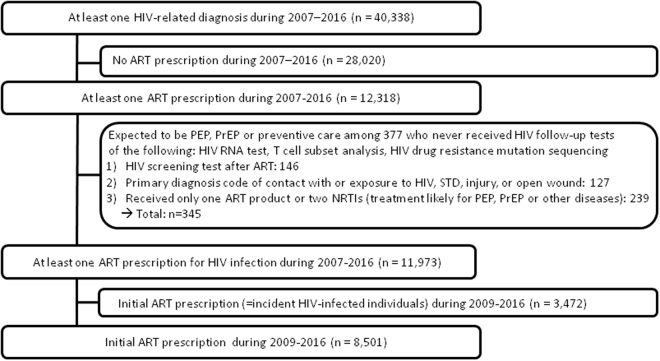
Flow chart of study population Abbreviations: ART, antiretroviral therapy; HIV, human immunodeficiency virus; PEP, post-exposure prophylaxis; PrEP, pre-exposure prophylaxis; STD, sexually transmitted disease.

The study population consisted of 8,501 HIV-infected individuals during 2009–2016 (Fig. 1). We identified 5,981 (70.4%), 798 (9.4%), 654 (7.7%), and 1,068 (12.6%) individuals in the four medication possession ratio (MPR) groups (≥95%, 80–95%, 50–80%, and <50%), respectively (Table 1). Men accounted for 92% of the study population. In both cases of AIDS defining illness and requiring prophylactic antibiotics, we observed the highest proportion of individuals in the MPR <50% group with statistically significant trend of increase as adherence gets worse. Most patients (93%) were insured through the NHI, with the remaining 7% being covered by the National Medical Aid (NMA) (p < 0.0001). In case of the year of diagnosis, the proportion of patients with adequate adherence of MPR ≥95% increased continuously from 2009 to 2016 with statistically significant trend (p < 0.0001). When long term follow-up loss (LTFUL) was defined as no record of a clinic visit for ART prescription for same or more than a year, 223 (20.9%) belonged in MPR <50% group while 16 (0.3%) were in MPR ≥95% group (p < 0.0001).

**Table 1:** Characteristics of newly diagnosed HIV-infected individuals by medication possession ratio, 2009–2016.

	Total (n = 8,501)	≥95% (n = 5,981)	80–95% (n = 798)	50–80% (n = 654)	<50% (n = 1,068)	*p* value^a^
Gender						
Men	7,824 (92.0)	5,560 (93.0)	734 (92.0)	595 (91.0)	935 (87.5)	<0.0001
Women	677 (8.0)	421 (7.0)	64 (8.0)	59 (9.0)	133 (12.5)	
Age						
0–19	225 (2.6)	143 (2.4)	29 (3.6)	23 (3.5)	30 (2.8)	<0.0001
20–29	2,135 (25.1)	1,548 (25.9)	198 (24.8)	192 (29.4)	197 (18.4)	
30–39	2,202 (25.9)	1,579 (26.4)	212 (26.6)	161 (24.6)	250 (23.4)	
40–49	2,001 (23.5)	1,386 (23.2)	197 (24.7)	149 (22.8)	269 (25.2)	
Over 50	1,938 (22.8)	1,325 (22.2)	162 (20.3)	129 (19.7)	322 (30.1)	
AIDS defining illness	1,655 (19.5)	1,114 (18.6)	168 (21.1)	125 (19.1)	248 (23.2)	0.001
Requiring prophylactic antibiotics	3,213 (37.8)	2,029 (33.9)	332 (41.6)	286 (43.7)	566 (53.0)	<0.0001
Comorbidity						
Diabetes	1,861 (21.9)	1,356 (22.7)	153 (19.2)	120 (18.3)	232 (21.7)	0.07
Hypertension	1,274 (15.0)	891 (14.9)	101 (12.7)	87 (13.3)	195 (18.3)	0.07
Malignancy	849 (10.0)	554 (9.3)	74 (9.3)	65 (9.9)	159 (14.9)	<0.0001
Psychiatric disorder	3,431 (40.4)	2,498 (41.8)	302 (37.8)	242 (37.0)	389 (36.4)	<0.0001
Viral hepatitis	2,196 (25.8)	1,608 (26.9)	201 (25.2)	165 (25.2)	222 (20.8)	<0.0001
Financial status						
NHI	7,878 (92.7)	5,648 (94.4)	737 (92.4)	582 (89.0)	911 (85.3)	<0.0001
National Medical Aid	623 (7.3)	333 (5.6)	61 (7.6)	72 (11.0)	157 (14.7)	
Hospital type						
Designated tertiary hospital	5,722 (67.3)	4,044 (67.6)	540 (67.7)	449 (68.7)	689 (64.5)	0.1
Others^b^	2,779 (32.7)	1,937 (32.4)	258 (32.3)	205 (31.3)	379 (35.5)	
Hospital region						
Metropolitan cities	6,442 (75.8)	4,516 (75.5)	613 (76.8)	515 (78.7)	798 (74.7)	0.8
Rural	2,059 (24.2)	1,465 (24.5)	185 (23.2)	139 (21.3)	270 (25.3)	
Diagnosed year						
2009	781 (9.2)	412 (6.9)	122 (15.3)	91 (13.9)	156 (14.6)	<0.0001
2010	866 (10.2)	483 (8.1)	120 (15.0)	102 (15.6)	161 (15.1)	
2011	965 (11.4)	568 (9.5)	136 (17.0)	98 (15.0)	163 (15.3)	
2012	981 (11.5)	620 (10.4)	122 (15.3)	91 (13.9)	148 (13.9)	
2013	1,098 (12.9)	777 (13.0)	96 (12.0)	88 (13.5)	137 (12.8)	
2014	1,251 (14.7)	936 (15.6)	99 (12.4)	79 (12.1)	137 (12.8)	
2015	1,237 (14.6)	1,018 (17.0)	63 (7.9)	56 (8.6)	100 (9.4)	
2016	1,322 (15.6)	1,167 (19.5)	40 (5.0)	49 (7.5)	66 (6.2)	
Long term follow-up loss^c^	477 (5.6)	16 (0.3)	30 (3.8)	208 (31.8)	223 (20.9)	<0.0001

Abbreviation: AIDS, acquired immune deficiency syndrome; HIV, human immunodeficiency virus; NHI, National Health Insurance.

^a^Mantel-Haenszel Chi-Square test to assess trends in the four MPR groups. ^b^Others of hospital type include public health centres, primary medical clinics, general hospitals. ^c^Individuals who had no clinic visit for antiretroviral treatment for same or more than a year.

### Hospital visit characteristics

In the high adherence group, means numbers of clinic visit for follow-up tests (HIV RNA quantification test, T cell subset analysis, HIV drug resistance mutation sequencing) or ART prescription were larger, and ART prescription days per visit was longer (p < 0.0001) (Table 2). The number of concomitant medication except ART was not different among the four groups. Although the proportion of individuals who visited emergency department at least once for any reason was not significantly different among the MPR groups, the number of hospital admission did show a significant difference, with higher frequency in the lower adherence group (*p* < 0.0001).

**Table 2:** Characteristics of hospital visits of HIV patients by medication possession ratio.

	≥95% (n = 5,981)	80–95% (n = 798)	50–80% (n = 654)	<50% (n = 1,068)	*p* value
Clinic visit for follow-up test^a^	13 ± 8.3	16 ± 8.1	15 ± 8.5	6 ± 5.3	<0.0001
Clinic visit for ART	19 ± 13.2	25 ± 13.4	22 ± 12.9	8 ± 8.3	<0.0001
ART prescription days per visit	67 ± 23.0	63 ± 19.7	53 ± 20.7	32 ± 20.2	<0.0001
Co-medication number^b^	1.2 ± 2.4	1.1 ± 2.6	1.3 ± 2.6	1.3 ± 3.4	0.4
Emergency visit	3,265 (54.6)	447 (56.0)	408 (62.4)	513 (48.0)	0.06
Hospital admission	4,409 (73.7)	629 (78.8)	528 (80.7)	930 (87.1)	<0.0001

Abbreviation: ART, antiretroviral therapy; HIV, human immunodeficiency virus.

^a^HIV RNA quantification test, T cell subset analysis, HIV drug resistance mutation sequencing. ^b^The maximum number of co-medication except ART divided by the number of observed months. Mean ± standard deviation in case of clinic visit, ART prescription days, and co-medication number. Number of patients (% in each group) in case of emergency visit and hospital admission.

### Risk factors for suboptimal ART adherence

Risk factors for low ART adherence were analysed by comparing the ≥95% MPR group to the remaining three groups using a multivariable logistic regression model (Table 3). Patients who required prophylactic antibiotics were more likely to exhibit inadequate adherence (Odds Ratio [OR] 1.7, 95% confidence interval [CI] 1.5–2.0), while AIDS defining illness did not show statistical significance after adjustment. When comparing age groups, being in their 20 s and same or over 50 s showed a significant association with suboptimal adherence than their 30 s (OR 1.6, 95% CI 1.1–2.4, and OR 1.4, 95% CI 1.2–1.7, respectively). Being supported by the NMA, a proxy for lower financial status, was also significantly associated with suboptimal adherence when compared to being supported by the NHI (OR 2.1, 95% CI 1.7–2.6). In contrast, patients with psychiatric disorders had a lower likelihood of suboptimal adherence (OR 0.62, 95% CI 0.41–0.92); the directionality of this association remained when behavioural disorders related to only depression or alcohol use were separately included in the model.

**Table 3:** Risk factors for low adherence (medication possession ratio <95%) among newly diagnosed HIV-infected individuals.

	**cOR**	**95% CI**	**aOR** ^**a**^	**95% CI**
Gender				
Male	1 (ref)		1 (ref)	
Female	1.8	1.5–2.2	1.6	1.3–2.0
AIDS defining illness	1.3	1.1–1.5		
Requiring prophylactic antibiotics	2.0	1.8–2.3	1.7	1.5–2.0
Age				
0–19	1.2	0.8–1.8	1.6	1.1–2.4
20–29	0.8	0.7–1.0	1.0	0.8–1.3
30–39	1 (ref)		1 (ref)	
40–49	1.2	1.0–1.5	1.1	0.9–1.3
50–	1.6	1.3–1.9	1.4	1.2–1.7
Comorbidity				
Diabetes	1.0	0.8–1.2		
Hypertension	1.3	1.1–1.6		
Malignancy	1.7	1.4–2.0	1.6	1.3–1.9
Psychiatric disorder	0.8	0.7–0.9	0.8	0.7–0.9
Viral hepatitis	0.7	0.6–0.8	0.8	0.6–0.9
Financial status				
NHI	1 (ref)		1 (ref)	
National Medical aid	2.6	2.1–3.1	2.1	1.7–2.6
Hospital type				
Designated tertiary hospital	1 (ref)		1 (ref)	
Others^b^	1.2	1.0–1.3	1.2	1.1–1.4
Diagnosed year				
2009	4.8	3.5–6.4	3.7	2.7–5.0
2010	4.3	3.2–5.9	3.8	2.8–5.1
2011	3.9	2.9–5.2	3.4	2.5–4.6
2012	3.4	2.5–4.6	3.2	2.3–4.3
2013	2.7	2.0–3.7	2.5	1.8–3.4
2014	2.3	1.7–3.2	2.3	1.7–3.1
2015	1.7	1.2–2.3	1.6	1.2–2.3
2016	1 (ref)		1 (ref)	

Abbreviation: AIDS, acquired immune deficiency syndrome; aOR, adjusted odds ratio; CI, confidence interval; cOR, crude odds ratio; HIV, human immunodeficiency virus; NHI, National Health Insurance; ref, reference.

^a^Adjusted for all the variables in the table in addition to hospital region and the maximum number of all co-medication except ART divided by the number of observed months. ^b^Others of hospital type include public health centres, primary medical clinics, general hospitals.

## Discussion

This study demonstrates the levels of adherence to ART in HIV-infected individuals in Korea. When the study population was stratified into four groups according to the MPR, 70.4% showed an adherence of ≥95%. Although this does not meet the UNAIDS target of 90%, it seems acceptable when compared to previous studies in other developed and developing countries^6,14–18^. In a study conducted in Malawi, 70% of patients had a MPR >90%, and a study conducted in another sub-Saharan African country reported that 52% of patients showed an adherence of >80% based on a pharmacy dispense records^6,14^. A meta-analysis of 27 studies from 12 sub-Saharan African countries and 31 North American studies revealed that 77% of HIV-infected individuals achieved adequate adherence based on the criteria used in the respective studies; in contrast, only 55% of HIV-infected individuals did in North American studies^15^. A study in France reported that 65.2% of HIV patients showed an MPR of >80%^16^. Last, a study conducted in the United States revealed an adherence of >90% in only 38% of the study participants^17^.

In this study, we showed that patients requiring prophylactic antibiotics, women, age under 19 or over 49 compared to 30–39, history of malignancy, lower socioeconomic status, visiingt clinics either than specialized tertiary teaching hospital, and being newly diagnosed in the years earlier than 2016 were at a higher risk of becoming less adherent. All costs for HIV treatment, including ART and outpatient or inpatient clinic care, are completely covered by the government in Korea. Nonetheless, this study showed that being insured by the NMA, an indicative of a lower socioeconomic status, was a risk factor for suboptimal adherence. The proportion of patients supported by the NMA among the entire HIV population was higher (7%) than that among the national population (3%), showing that HIV-infected individuals tend to have a lower socioeconomic status in general. Data on whether socioeconomic status is important for ART adherence have been inconsistent^19,20^. In addition to the cost of medical treatment, labour loss time and transportation problems are barriers to adherence^20,21^. As a lower socioeconomic status is frequently accompanied by a lower educational level, an incomplete understanding of treatment importance or simply forgetting taking medication is another barrier to adherence^21,22^. However, a recent survey among men who have sex with men in Korea revealed that education or financial status was not a barrier for the intention to take HIV screening test^23^. In addition, we found that patients who visit hospitals either than government designated tertiary teaching hospitals were at higher risk of low adherence compared to those who visit designated ones. Since there is no difference in financial barrier among medical institutions for ART, patients with high motivation of treatment tend to visit government designated tertiary teaching hospitals. This implies more interest in the care of HIV-infected individuals outside the range of tertiary hospitals, both the attention of the clinicians and policy makers regarding lower level of medical institutions.

In terms of comorbid conditions, having a psychiatric disorder and viral hepatitis were both negatively associated with suboptimal adherence. When we analysed the diagnoses within psychiatric disorder by subcategories, the majority of diagnoses (45%; data now shown) were depressive illnesses. The direction of the association did not change when only depression (instead of psychiatric disorders as a whole) was included in the model. This result is not in agreement with previous studies, which reported depression itself as a risk factor for lower adherence^8,24,25^. However, compared to the prevalence of depression of 22–32% among HIV-infected individuals in a previous report^26^, the prevalence in the current data set is similar. Considering the social stigma associated with depression^27^, it is possible that HIV patients who had a better relationship with their physician were more likely to report their depressive symptoms than those with a poorer relationship. In addition, ART adherence study of HIV and viral hepatitis C (HCV) co-infected individuals also revealed that PDC >95% group had higher proportion of HCV treatment using the 2005–2007 Medicaid claims data^28^. Overall, improved patient-clinician relationships could enhance ART adherence^29^. Counselling and more in-depth communications between patients and clinicians can facilitate the detection of comorbidities such as depression, improve ART adherence and the treatment outcome of comorbid conditions.

While HIV-infected individuals in Korea are mostly male, female was a risk factor for suboptimal adherence. This is in line with other studies where in one study, women had 1.5 times greater incidence of non-adherence initiating ART in a 2001–2002 cohort in Brazil^30^, and in another study, female gender had a lower likelihood (OR 0.7) of being 95% adherent in Canada^31^. Also in Korea where HIV is commonly known to transmit through sexual contact, women tend to be reluctant to expose themselves. However, higher proportion of female also explains the higher risk of low adherence among those with malignancy history since 49% of whose with malignancy were female. Among 8,501 total study population, 54 had a primary diagnosis of malignancy when initial ART treatment was prescribed, and the type and frequency were the following: stomach: 1, colon: 1, anus: 1, liver: 7, biliary tract: 1, pancreas: 1, lung: 1, Kaposi sarcoma: 2, soft tissue: 1, breast: 1, kidney: 1, bladder: 1, brain: 2, Hodgkin lymphoma: 1, all other types of lymphoma: 30, leukaemia: 2. Even though there are identified challenges for cancer patients to initiate ART such as drug-drug interactions, overlapping toxic effects, or immunodeficiency associated with malignancy, most ART regimen can be implemented. Therefore, cancer history should not be a barrier for ART treatment and the cooperation of both specialists of cancer and infectious disease should be encouraged while most of the HIV-infected individuals in Korea visit highly specialized tertiary hospitals.

The main limitations of this study comes from the reimbursement-based claims databases which generally contain only the information provided by health services and lack some demographic data such as education or clinical manifestations such as viral load or CD4+ T cell values. In Korea, however, all HIV-related medical care is reimbursed; thus, we assume that all services that HIV patients received were fully recorded in the database. Even though the values of the follow-up tests were not available, we analysed the frequency of relevant tests and used the prescription history of prophylactic antibiotics as a proxy for CD4+ T cell counts <200 cells/mm^3^ as prophylactic antibiotics are prescribed in those patients with advanced HIV infection. Another limitation is the potentially inaccurate recruitment of HIV-infected individuals. Since we selected HIV-infected individuals starting from ART receivers, those who were diagnosed with HIV infection but never visited healthcare could have been excluded while those who received ART for other purpose could have been falsely included.

However, the portion of undetected population is expected to be very low in a country with a very low financial barrier to ART treatment, and when the number of incident HIV patients from this database was compared to the actual national report, the number was slightly higher in this database. More importantly, we excluded those who seemed to have received ART for PEP, PrEP, or other reasons using various ways. Last, we may not have detected all cases of deaths by using the ICD-10 codes, and this could have resulted in a lower adherence than real since the time after one is deceased was counted as LTFU. However, this was the only information available, and among a population of 20–49 consisting of 75%, this was relatively a minor issue.

In previous studies, adherence to ART was assessed in retrospective or prospective cohorts, which demands a huge financial burden and effort. The database of KCDC is the number of reported cases and these individuals are not periodically monitored by the KCDC, but by individual clinics when those individuals seek healthcare facilities. This study used claims data to analyse the nationwide adherence to ART with minimum effort and revealed comparable findings to previous studies, indicating a potential use of NHI database in ART adherence monitoring in a national level.

## Conclusion

The current policy for HIV control is more focusing on improving the continuum of HIV care from diagnosis to viral suppression. In particular, good medication adherence is one of essential factors to achieve optimal HIV care. However, our results showed that only 70.4% of HIV-infected individuals had adequate adherence to ART and there were modifiable risk factors affecting low adherence. Based on our results, health authority should take into consideration of how to establish sustainable monitoring system at national level using the NHI system for HIV care and how to increase the medication adherence for population with risk factors.

## Methods

### Data source

In Korea, 97% of the total population is registered in the obligatory NHI, and the remaining 3% (those with the lowest socioeconomic status) are supported by the NMA. All medical utilisation within the NHI is monitored for reimbursement, and all use of healthcare with the purpose of treating a medical condition is reimbursed in various proportions. The NHI claims database contains retrospective cohort data on basic information on the patients’ sociodemographic characteristics and visits to medical institutions as well as precise information on their diagnoses, prescriptions or diagnostic procedures and the characteristics of the medical clinics they visited^32^. HIV-related medical fees, not only those for ART but also those related to comorbidities relevant to HIV infection, are all supported by the NHI in Korea. Therefore, information on medical clinic visits, including data on prescriptions and diagnostic procedures can be obtained for both outpatient clinics and hospital admissions for all HIV-infected individuals.

### Study population

Of the nationwide NIH cohort of 50 million individuals, 40,338 individuals received an HIV infection diagnosis (ICD-10 codes B20-24) at least once during 2007–2016 (Fig. 1). Among these, 12,318 individuals had at least one ART prescription during the same period and among these ART receivers, 377 never went through any of the HIV follow-up tests of the following: HIV RNA quantification test, T cell subset analysis, HIV drug resistance mutation sequencing. Among these 377 individuals, 345 individuals who were expected to have received ART for post-exposure prophylaxis (PEP), pre-exposure prophylaxis (PrEP) or other preventive care were excluded. In specific, there were three categories of these excluded individuals. First, those who received HIV screening test after ART prescription were excluded since this implies that HIV infection was not confirmed after former ART prescription. Second, if there were primary diagnosis code of the following together with ART prescription, ART were expected to have been prescribed for other purpose: contact with or exposure to HIV, occupational exposure to risk factor, sexually transmitted diseases such as syphilis, major injury with bleeding, or open wound. Third, those who received only one ART product or two nucleoside analogue reverse transcriptase inhibitor (NRTI)s were likely to have received ART for other purpose than HIV infection treatment considering the number of ART products. The day of first ART prescription was regarded as the day of initial diagnosis of HIV infection, as well as the index date of cohort recruitment for adherence study. In order to select only incident HIV-infected individuals for follow-up of ART prescription from the database of 2007-2016, we excluded those who had ART prescription before 2009 and recruited only those who had first ART prescription after two years of window period, resulting in 8,501 final study population of incident HIV-infected individuals during 2009–2016.

### Definitions

The characteristics of the study population was accessed only from the database before the time of one's initial ART, which was used as the time of initial HIV infection diagnosis and cohort index date. Therefore, the risk factors evaluated in this analysis could overcome reverse causation. For example in case of comorbidity, the existence of certain comorbid condition was searched within the records of one’s many clinic visits only before the day of initial visit for ART. The time of the initial ART prescription, a substitute for initial HIV diagnosis, was used to determine age, health insurance status (used as a proxy for socioeconomic status), and region/type of the visited hospital. A person with AIDS was defined as an individual who received at least one diagnosis of an AIDS-defining illness as defined by the CDC^33^. Individuals who were prescribed with prophylactic dosage of trimethoprin/sulfamethoxazole or dapsone for the prevention of pneumocystis pneumonia were defined as ‘Requiring prophylactic antibiotics’, which could indicate CD4+ T cell counts <200 cells/mm^3^. As infectious disease specialists in tertiary teaching hospitals mainly treat HIV-infected patients in Korea, the types of hospitals were categorized as designated tertiary hospital *vs*. others. Among tertiary hospitals, the government entitles hospitals with high proportion of severe patients as designated tertiary hospital every 3 years. When grouping hospitals by their region, we defined metropolitan cities as cities designated as such by the government which is a large part a classification by its’ economic size including the capital city. In the comparisons of the clinical characteristics of HIV patients by MPR group, we analysed the frequency of visits to the emergency department and that of hospital admissions (any kind of visit or admission regardless of HIV infection). Hypertension included primary hypertension, and hypertensive heart or renal diseases (ICD-10 codes I10–13) but not secondary hypertension. Malignancy included all kinds of neoplasm including malignant or benign ones (ICD-10 codes C00–D48), and viral hepatitis included all viral hepatitis including acute or chronic hepatitis B or C. Psychiatric disorders included all mental or behavioural conditions such as dementia, mood disorders including depression, and unspecified mental disorders (ICD-10 codes F00-99). In our analysis of risk factors for suboptimal adherence, we adjusted for the number of all prescribed medications including medication for conditions other than the HIV infection: We divided this number by the number of months of observation to reflect the total duration of observation. Medications included only reimbursed medications (excluded over-the-counter medications).

### Adherence measurements

The start of observation period was the first ART fill date, and the end was one of the two cases: until the last refill date for those who never returned to care after loss to follow-up (LTFU), or the last day of 2016 for those who were still under follow-up. The MPR was calculated as the sum of the days of treatment supplied for all ART prescriptions filled, from the first round of ART until the last day of 2016, divided by the number of days during that same time period. Since we were unable to differentiate between regular clinic visits with long gaps and infrequent visits with shorter gaps before or after a LTFU using the MPR, the pattern of long term follow-up loss (LTFUL) was analysed, which was defined as no medical clinic visit for ART for same or more than a year. Instead of applying various indices such as retention or LTFU to measure the patterns of clinic visits, as used in a previous study^34^, we utilized a single measurement, the MPR, to analyse adherence for the following reasons. First, the proportion of LTFUL was less than 6% and second, among these patients, the majority had the gap in the middle of care, not in the end. Last, among several methods of describing adherence such as retention to care or persistence, the proportion of days with ART treatment was considered as the most important factor for the management of HIV-infected individuals and of being of the highest interest for clinicians by the authors.

We used the MPR instead of the proportion of days covered (PDC) as HIV patients generally receive various different ART agents at single visit, covering the same period at each visit, which was also confirmed from the database. Moreover, as the intake of each ART agent could not be checked individually, it was not possible to use information of each agent within multiple drug regimens. As we recruited incident HIV-infected individuals, we assumed that they were all on their first line of ART. During the study period, the first regimen recommended by the clinicians included two nucleoside analogue reverse transcriptase inhibitor (NRTI) backbones plus one of the following: protease inhibitors, non- nucleoside analogue reverse transcriptase inhibitors, or integrase strand transfer inhibitors, as recommended by international guidelines^35^. For the NRTI backbone, the preferred agents in the guideline were tenofovir/emtricitabine and abacavir/lamivudine.

### Statistical analysis

Analysis of variance (ANOVA) was used to compare the means of the four MPR groups (≥95%, 80–95%, 50–80%, and <50%). For continuous variables, post-hoc analysis was conducted with the Tukey method, with a *p* value 0.001 considered significant, to test difference between the ≥95% MPR group and the other groups. The Chi-Square test was used to test the statistical significance of the differences between the groups. We applied Mantel-Haenszel Chi-Square test to assess trends in the four MPR groups; moreover, a pairwise comparison was performed between the ≥95% MPR group and the remaining 3 groups. Odds ratios (ORs) for suboptimal adherence (MPR <95%) were calculated using a multivariable logistic regression model with a stepwise option of an entry limitation with a *p* value of 0.05. To consider different lengths of observation periods and changes in treatment guidelines or available regimens on the market, the year of diagnosis was included as an adjusting variable. The final model was verified as adequate and the concordance statistic estimate (c) was 0.685 with a concordance of 68%. SAS Enterprise Guide, version 6.1 (SAS Institute, Inc., Cary, NC, USA) was used for all analyses.

### Ethics statement

The institutional review board of the Seoul National University Boramae Medical Centre (IRB No. 07-2017-8/052) approved this study. The board waived informed consent due to the use of an existing secondary database.
